# Single cell‐derived clonally expanded mesenchymal progenitor cells from somatic cell nuclear transfer‐derived pluripotent stem cells ameliorate the endometrial function in the uterus of a murine model with Asherman’s syndrome

**DOI:** 10.1111/cpr.12597

**Published:** 2019-03-21

**Authors:** Sung‐Min Jun, Mira Park, Ji Yoon Lee, Sookyung Jung, Jeoung Eun Lee, Sung Han Shim, Haengseok Song, Dong Ryul Lee

**Affiliations:** ^1^ CHA Advanced Research Institute Seongnam Korea; ^2^ Department of Biomedical Science CHA University Seongnam Korea

**Keywords:** angiogenesis, Asherman’s Syndrome, human pluripotent stem cells, mesenchymal progenitor cells, somatic cell nuclear transfer

## Abstract

**Objectives:**

Because primary mesenchymal progenitor cells (adult‐MPCs) have various functions that depend on the tissue origin and donor, de novo MPCs from human pluripotent stem cells (hPSCs) would be required in regenerative medicine. However, the characteristics and function of MPCs derived from reprogrammed hPSCs have not been well studied. Thus, we show that functional MPCs can be successfully established from a single cell‐derived clonal expansion following MPC derivation from somatic cell nuclear transfer‐derived (SCNT)‐hPSCs, and these cells can serve as therapeutic contributors in an animal model of Asherman's syndrome (AS).

**Materials and methods:**

We developed single cell‐derived clonal expansion following MPC derivation from SCNT‐hPSCs to offer a pure population and a higher biological activity. Additionally, we investigated the therapeutic effects of SCNT‐hPSC‐MPCs in model mice of Asherman's syndrome (AS), which is characterized by synechiae or fibrosis with endometrial injury.

**Results:**

Their humoral effects in proliferating host cells encouraged angiogenesis and decreased pro‐inflammatory factors via a host‐dependent mechanism, resulting in reduction in AS. We also addressed that cellular activities such as the cell proliferation and population doubling of SCNT‐hPSC‐MPCs resemble those of human embryonic stem cell‐derived MPCs (hESC‐MPCs) and are much higher than those of adult‐MPCs.

**Conclusions:**

Somatic cell nuclear transfer‐derived‐hPSCs‐MPCs could be an advanced therapeutic strategy for specific diseases in the field of regenerative medicine.

## 1 INTRODUCTION

It is now accepted that human pluripotent stem cells (hPSCs), such as embryonic stem cells (ESCs) and reprogrammed stem cells from somatic cells using Yamanaka factors or somatic cell nuclear transfer (SCNT), will be available as cell sources in regenerative medicine.^1^, ^2^, ^3^, ^4^, ^5^ Recently, we successfully established SCNT‐derived human PSCs (SCNT‐hPSCs) using patient fibroblasts^4^, ^6^ and have constantly developed the protocol to differentiate several lineage cells, including mesenchymal progenitor cells (MPCs), for application in cell therapy.

MPCs originate from various tissues and are regarded as promising therapeutic cell sources, findings that have already been shown in regenerative medicine and clinical trials.^7^, ^8^, ^9^, ^10^ Although the functions of MSCs are well addressed in diverse diseases, some perils of MPCs, such as rapid cell senescence in vitro, individual variations of donors, and non‐replenishment, remain to be overcome. To resolve these limitations mentioned above, hPSCs have emerged as a valuable alternative, and their therapeutic effects have been continuously reported in regenerative medicine. Particularly, rare genetic diseases that have maternally inherited mitochondrial DNA mutations, such as sideroblast anaemia and Parkinson's disorders with chromatin decondensation, required novel tools, such as SCNT‐hPSCs, to study their mechanism or develop clinical treatments. Because oocytes produce many proteins involved in the modulation of chromatin decondensation and mitochondria, SCNT‐hPSCs may provide some clues to overcome the demerits in the concept of therapeutic cloning.^11^, ^12^, ^13^, ^14^, ^15^ Additionally, SCNT‐hPSCs with a low risk of immune rejection is now the subject of interest, likely to be further applied in clinical trials.

In this study, we developed single cell‐derived clonal expansion following MPC derivation from SCNT‐hPSCs to offer a pure population and a higher biological activity. Additionally, we investigated the therapeutic effects of SCNT‐hPSC‐MPCs in Asherman's syndrome (AS), which is characterized by synechiae or fibrosis with endometrial injury, often leading to infertility. A proliferation of endometrial cells should be proceeded to treat AS. However, treatment is difficult due to the intricate machinery of basal layer production or loss of stem cells in the endometrium.^16^, ^17^ In this regard, MPCs could be a possible candidate to treat fibrosis of AS because they contain paracrine properties that encompass both angiogenic and anti‐inflammatory effects. In the present study, we addressed, for the first time, that single cell‐derived clonally expanded SCNT‐hPSC‐MPCs (SCNT‐hPSC‐MPC‐SCDs) showed increased cell numbers with stable population doublings (PDs) and no teratomas. These cells contributed to reduction in fibrosis in the AS model, resulting in implantation via promoted angiogenesis. Moreover, these cells show functional resemblance to hESC‐MPCs. Thus, the successful generation of functional SCNT‐hPSC‐MPCs can promote therapeutic advances using the novel alternative in regenerative medicine.

## 2 MATERIALS AND METHODS

### 2.1 Culture of human PSCs and differentiation into MPCs

**Figure 1:**
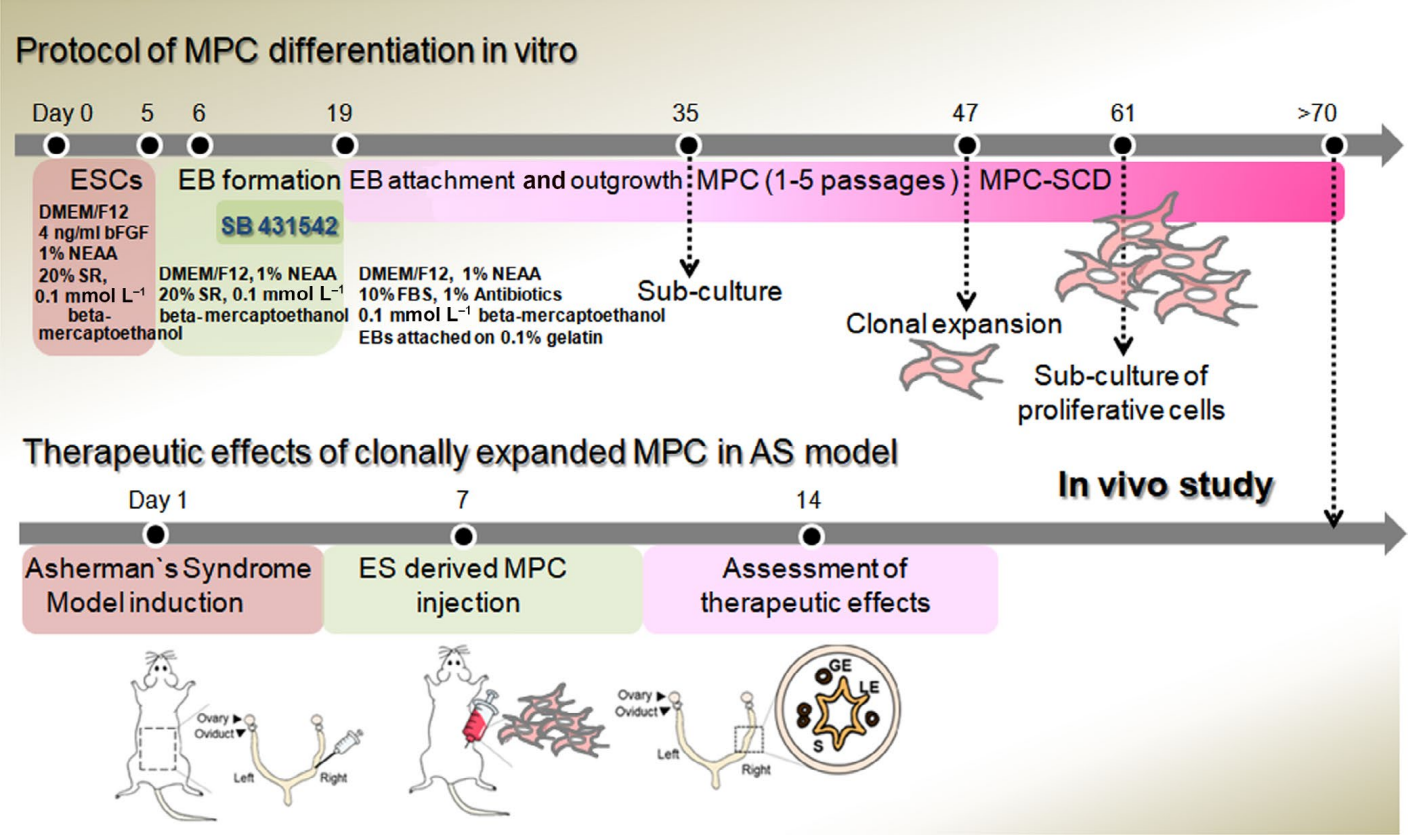
Schematic diagram describing the protocol for differentiation of somatic nuclear transfer‐derived human pluripotent stem cells into mesenchymal progenitor cells (SCNT‐hPSC‐MPCs). The formation of embryonic bodies (EBs) from PSCs was performed using SB431542, and then, EBs were reseeded in culture dishes for 28 d; the resultant cells are referred to as MPCs. Using MPCs detached in single‐cell suspension, single cell‐derived MPCs were clonally expanded in culture plates for at least 23 d. To examine the functions of the generated SCNT‐hPSC‐MPCs, their in vitro cellular activity was evaluated in confluent MPCs, and cells were applied to the Asherman's syndrome (AS) in vivo model. After cell transplantation in the uterus of the AS model, the assessment of the therapeutic effects was carried out at day 7

All experiments were performed under authorization from the Institutional Review Board for Human Research at the CHA University, Seongnam, Korea and the National IRB board regarding the research using hESCs. Human PSCs, including conventional ESCs (CHA‐hES15) and SCNT‐PSCs (CHA‐hNT5), were plated at 1 × 10^5^ cells per cm^2 ^onto mitotically inactivated mouse fibroblasts (MEFs) in DMEM/F12 medium supplemented with Knockout Serum Replacement (10% KSR; Invitrogen) and bFGF (4 ng/mL; Invitrogen) and were maintained as described previously. The characteristics of SCNT‐PSCs (CHA‐hNT5) were provided in Figure S2. As an experimental control, bone marrow (BM)‐MPCs (PT2501, Lonza, Walkersville, MD) were used. During the differentiation of MPCs, the medium was changed every 3 days. For details, a schematic illustration of the differentiation protocol is provided in Figure 1.

### 2.2 Cell proliferation assay

For the analysis of cell growth during long‐term culture, cells were maintained in expansion culture as follows: 5 × 10^4^ cells per well were plated in 12‐well culture dishes, and the cells were counted in triplicate at least until 75 days after plating. The PDs and doubling time between cell passages were evaluated as previously described.^18^ The single cell‐derived MPCs were expanded and/or then combined to analyse their doubling time.

### 2.3 Induction of a mouse model of AS

Protocols for the use of animals in all experiments were approved by the Institutional Animal Care and Use Committee of CHA University (IACUC), and animal procedures were performed in accordance with approved guidelines and regulations (IACUC approval number 170137). Regarding the AS mouse model, 8‐week‐old ICR female mice werepurchased from KOATECH (Pyeontaek, Gyeonggi, Korea) and were used to induce a traumatized AS model. After anaesthesia by avertin, a vertical incision was made in the abdominal wall, and the uterus was exposed. A small incision was made in each uterine horn at the utero‐tubal junction, and the horn was traumatized in a standardized fashion using a 30 gauge needle inserted through the lumen, rotated and withdrawn 10 times.^19^ Forty‐eight mice with AS were randomized to various experiments to investigate the therapeutic potential of SCNT‐hPSC‐MPCs in injured uteri with fibrosis. For cell transplantation, cells (1 × 10^6^) were directly injected into uterus with twice time for 2 weeks, after anaesthetizing the untested mice for additional oestrus cycle. In the first model study for AS, experimental groups were followed: Sham group: sham operation only, n = 4; AS only group: induction of trauma only, n = 4; hBM‐MPC group: induction of trauma and then injection of BM‐MPCs, n = 6; CHA‐hESC15‐MPC‐SCD group: induction of trauma and then injection of single cell‐derived clonally expanded conventional ESC (CHA‐hESC‐15)‐derived MPCs, n = 6; and CHA‐hNT5‐MPC‐SCD group: induction of trauma and then injection of single cell‐derived clonally expanded SCNT‐ESC (CHA‐hNT5)‐derived MPCs, n = 6. In the next implantation study, female mice with uterine horn AS‐induced were mated with fertile male mice at 7 days after cell transplantation: Sham/AS group: one uterine horn was sham‐operated and the other was trauma‐induced only, n = 4; AS/hBM‐MPC group: one uterine horn was trauma‐induced only and the other was trauma‐induced and then injected with BM‐MPCs, n = 6; AS/CHA‐hESC15‐MPC‐SCD group: one uterine horn was trauma‐induced only and the other was trauma‐induced and then injected with single cell‐derived clonally expanded CHA‐hESC‐15‐derived MPCs, n = 6; and AS/CHA‐hNT5‐MPC‐SCD group: one uterine horn was trauma‐induced only and the other was trauma‐induced and then injected with single cell‐derived clonally expanded CHA‐hNT5‐derived MPCs, n = 6.

### 2.4 Statistical analysis

All results are presented as the mean ± SE. Statistical analyses were performed using the Mann‐Whitney *U *test for comparisons between two groups and the Kruskal‐Wallis ANOVA test for >2 groups. Values of *P < *0.05 were considered to denote statistical significance. GraphPad Prism ver. 4 software (GraphPad Software, La Jolla, CA, USA) was used for statistical analysis.

## 3 RESULTS

### 3.1 Effective differentiation and validation of MPCs from human PSCs

**Figure 2:**
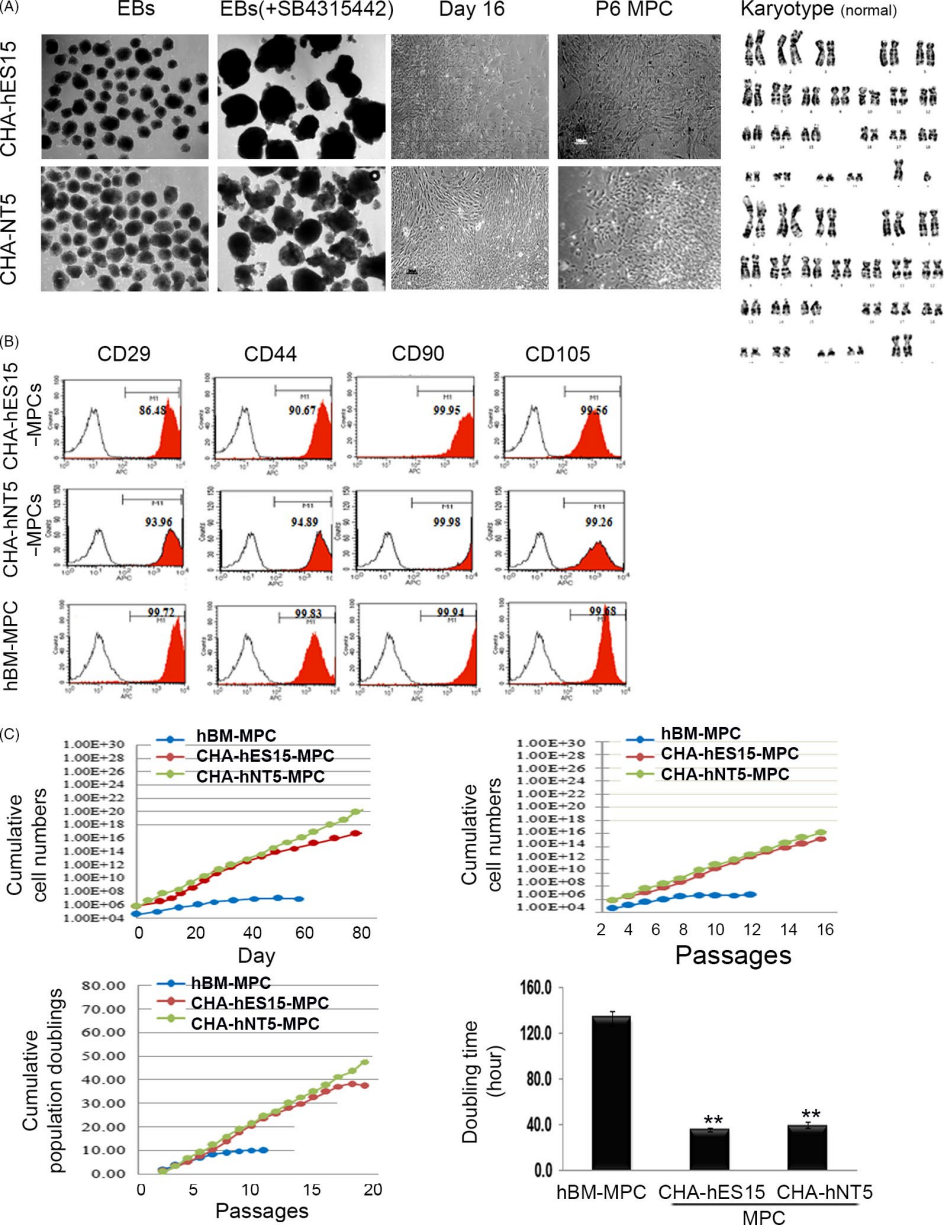
Sequential differentiation of hPSCs into MPCs. (A) The morphology of PSCs in terms of differentiation showed that fibroblast‐like MPCs were successfully produced under proper culture conditions at day 16 and at passage 5. MPCs displayed a normal karyotype, suggesting no senescence due to differentiation. Magnification, ×40 (B) FACS analysis showed that the markers for MPCs, CD29, CD44, CD90 and CD105 were abundantly increased in CHA‐hES15‐MPCs and CHA‐hNT5‐MPCs, suggesting a phenotypical resemblance between ES cells and SCNT cells. (C) The cellular activities of both hPSC‐MPCs revealed that exceeding proliferation by day and passages, and high population doublings were increased compared with those of adult BM‐MPCs. Senescence of BM‐MPCs occurred at approximately passage 12, while cell senescence in hPSC‐MPCs occurred after passage 20 with a marked increase in cell number, suggesting the possibility of overcoming the weakness of adult BM‐MPCs. The doubling times of hPSC‐MPCs were significantly decreased. The data are presented as the means ± SE from at least three experiments. Asterisks depict statistically significant differences compared with those of adult BM‐MSCs (**P < 0.05).

The generated CHA‐hES15 and CHA‐hNT5 cells from our laboratory were used in the present study (Figure S1).^4^, ^20^ Because the TGF‐beta inhibitor SB431542 is a known inducer that can reproduce mesodermal lineage cells,^21^ it was mainly used to encourage the commitment of the mesodermal lineage during EB formation. To effectively acquire the MPC population, two phased protocols were explored: (a) MPC stage via mesodermal lineage‐committed EB and (b) single MPC‐derived clonally expanded MPC stage. The attached EBs were allowed initially to outgrow in culture plates for 16 days and were sub‐cultured for expansion, when confluent cells reached approximately 80%. Thereafter, the cells were further cultured in standard medium for 12 days (approximately 5‐6 passages). The morphology of human PSC‐derived MPCs (hPSC‐MPCs) exhibited fibroblast‐like shapes similar to that of BM‐MPCs. The karyotype of differentiated MPCs was normal (Figure 2A), suggesting stable differentiation without chromosome alteration. Because adult BM‐MPCs are widely used as a gold standard for therapy, we estimated cell surface marker expression, differentiation into multilineage cells and PDs in the generated hPSC‐MPCs compared with those in BM‐MPCs. The immunophenotype of hPSC‐MPCs was strictly addressed using CD29, CD44, CD90 and CD105. All markers for MPCs, according to our data, clearly showed high frequencies over 90%, even CD24, which was rarely expressed in MPCs (data not shown). CD24 negativity is regarded as a marker for MPCs with CD105‐positive phenotype.^22^, ^23^ The expression of these markers for MPCs was definitively similar to that of BM‐MPCs (Figure 2B). Additionally, the generated hPSC‐MPCs were differentiated into multilineage cells involved in adipogenesis, osteogenesis and chondrogenesis (Figure S2). We next investigated the propagation of hPSC‐MPCs by counting the cell number, PDs and doubling time between cell passages compared with those of hBM‐MPCs. As shown in Figure 2C, we found that CHA‐hES15‐MPCs and CHA‐hNT5‐MPCs had an exponential proliferation index and underwent more PDs than hBM‐MPCs. Regarding the proliferative status in terms of passage, an exorbitant proliferation of cells was detected in both hPSC‐MPCs compared with that in hBM‐MPCs, which rapidly reached a plateau in the proliferation curve. Consistent with previous data,^24^ hBM‐MSCs were gradually decreased or sustained their cell expansion after 10 passages, suggesting cell senescence without cell division. However, hPSC‐MPCs still demonstrated sharply increased cell proliferation with no alteration in the cell morphology (Figure 2A,C), suggesting a rejuvenated status of hPSC‐MPCs. Additionally, we evaluated cell growth in long‐term culture by measuring the cell number to count PDs. Similar to cell expansion in long‐term culture, hPSC‐MPCs underwent 39‐50 PDs over 20 days, while hBM‐MPCs only accumulated 10 PDs with no additional expansion. The doubling time was significantly shorter in hPSC‐MPCs than in BM‐MPCs (3.33‐folds), suggesting rapid expansion over short periods (Figure 2C, right low graph).

### 3.2 Clonal expansion of MPCs from single MPCs and their characteristics: clonality, surface markers, euploidy and proliferation

**Figure 3:**
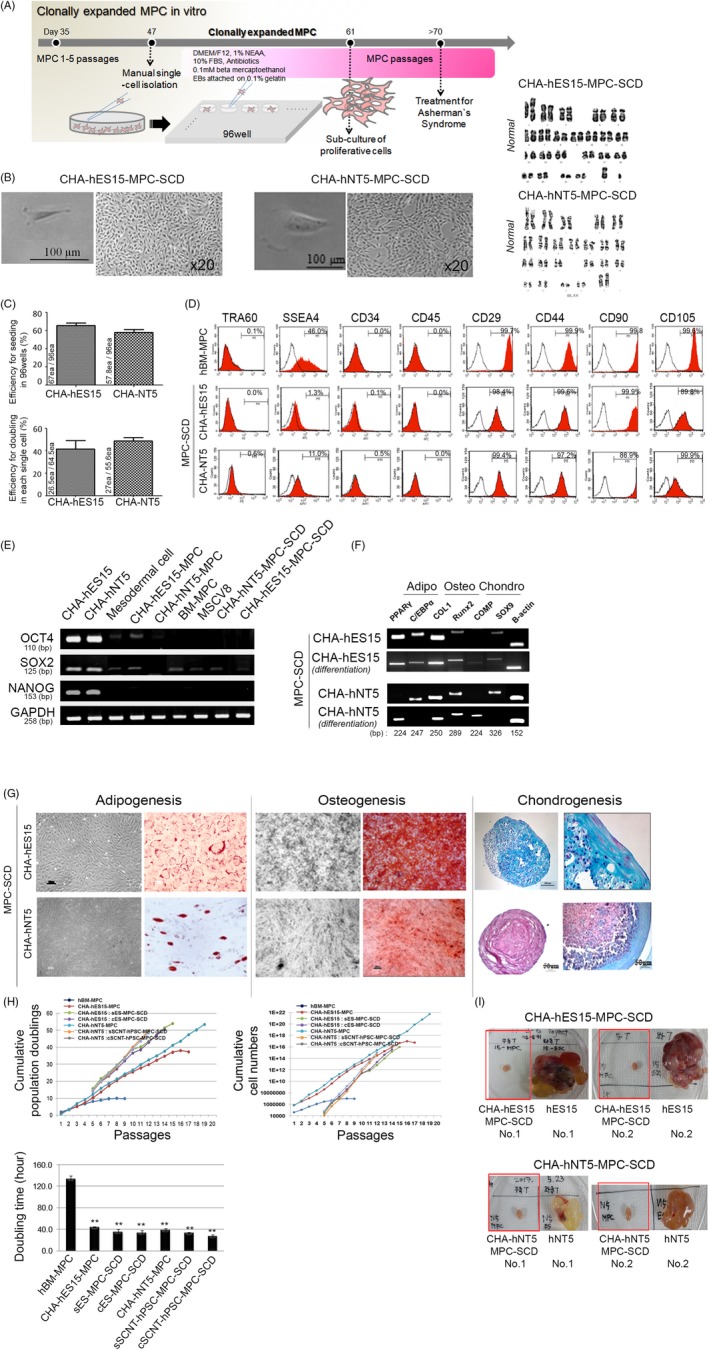
Generation of stable single cell‐derived clonally expanded SCNT‐hPSC‐MPCs (SCNT‐hPSC‐MPC‐SCDs). (A) Schematic diagram of single cell‐derived clonally expanded MPCs for the treatment of Asherman's syndrome (AS). (B) The morphology of MPCs derived from both hPSC‐MPCs is similar to that of adult tissue‐derived MPCs. After 5 passages, cells had a normal karyotype, implying no senescence. Bars, 100 μm. Magnification, ×20. (C) No difference between hESC‐ and SCNT‐hPSC‐MPCs was detected in the efficiency of seeding and doubling. When single MPCs were manually seeded into one well of a 96‐well plate, the survival rate excluded from seeding damage and capacity for proliferation of survived single MPCs were very similar, suggesting the resemblance of SCNT‐hPSC to hESCs. (D) FACS analysis showed that the markers for MPCs, CD29, CD44 and CD105 were highly increased in both hPSC‐MPC‐SCDs but not in stem cell or hematopoietic markers, suggesting phenotypical maturation. (E) Genes displaying stemness were rarely detected and were gradually decreased upon differentiation. (F) In multiple lineage differentiation, the *PPARγ, C/EBPα *for adipocytes*, COL1, RUNX2 *for osteocytes and *COMP*, *SOX9 *for chondrocytes in hPSC‐MPCs were detected by differentiation. All transcripts were expressed in multilineage cell differentiation. (G) To confirm their maturation, hPSC‐MPCs using standard differentiation conditions for adipogenesis, osteogenesis and chondrogenesis were differentiated; however, adipogenesis was not fully differentiated in MPC status, suggesting the not full maturation of hPSC‐MPCs into adipocyte, osteocyte and chondrocyte. (H) The cellular activities of both hPSC‐MPC‐SCDs displayed that proliferation by day and passage and high population doublings were increased. PDs and cumulative cell number were presented in terms of single cell preparation at passage 5. The data are presented as the means ± SE from at least three experiments. Asterisks depict statistically significant differences compared with adult BM‐MPCs (**P < 0.05). (I) No teratomas in both SCNT‐hPSC‐MPC‐SCDs were observed, implying safety in the clinic applications

To guarantee high purity and no teratomas of hPSC‐MPCs from undifferentiated stem cells, we selected a single MPC from detached cell suspension and further cultured single MPCs to induce the clonal expansion of MPCs without cell heterogeneity. In brief, differentiated and expanded MPCs were sub‐cultured and manually subjected to single cell seeding in culture dishes (0.8 cells per well in a 96‐well plate). The reseeded single MPCs (hPSC‐MPC‐SCDs) were expanded and monitored every other day for 14 days. The expanded cells, representing the progeny of 1 clone, from each well were reseeded into 1 well of a 4 well culture dish. These MPCs were continuously cultured in a larger culture dishes until 5 passages and then underwent the verification for MPC characters and transplantation into the uteri of AS model mice. A schematic diagram provides the procedure for clonal expansion of MPCs from a single cell (Figure 3A). Figure 3B shows the cell morphology in single cells and confluent expanded cells with a normal karyotype. Of the wells verified to contain a single cell (Figure 3B), the survival and proliferation were tested in the cells of selected wells. We found that no difference in the cellular capacity was detected in hPSC‐MPC‐SCDs with 67.0 ± 4.0% in CHA‐hES15‐MPCs and 57.8 ± 3.4% in CHA‐hNT5‐MPCs in the frequency of survival cells. Regarding the doubling efficiency, 41.5 ± 7.5% in CHA‐hES15‐MPCs and 48.8 ± 2.8% in CHA‐hNT5‐MPCs were similarly detected (Figure 3C). To analyse the characteristics, all hPSC‐MPC‐SCDs underwent FACS analysis to confirm their phenotype for MPCs. Rare expression of CD34, CD45, TRA60 and SSEA4 was detected due to the restriction of their expression in haematopoietic or stem cells as previously described.^25^ CD29, CD44 and CD105 were positively expressed in differentiated MPCs from both hPSC‐MPC‐SCDs, demonstrating the normal properties of MPCs. Tra60 and SSEA4 were hardly detected in both hPSC‐MPC‐SCDs, while SSEA4 was expressed in hBM‐MPCs at 46.0%, similar to that reported previously (Figure 3D).^26^ In the case of pluripotency genes, the expression levels of *OCT4, SOX2* and *NANOG* were highly reduced during differentiation (Figure 3E). The precursor genes for multilineage differentiated cells, precursor markers for each lineage, *C/EBPα, RUNX2 and SOX9,* were clearly expressed in both hPSC‐MPC‐SCDs. Maturation‐related genes *PPARγ* for adipocytes, *COL1 *for osteocytes and *COMP* for chondrocytes were detected following differentiation (Figure 3F). Next, we performed confirmative differentiation for lineage cells from SCNT‐hPSC‐MPC‐SCDs, and we found strong adipogenesis, osteogenesis and chondrogenesis in both hPSC‐MPC‐SCDs (Figure 3G).

In our first data, we found that cell proliferation and PDs of the SCNT‐hPSC‐MPC‐SCD were remarkably discriminated from BM‐MPCs (Figure 3H and Figure S3). Furthermore, we investigated whether the capacity for clonal propagation and differentiation of hPSC‐MSC‐SCDs can synergistically increase when MPCs obtained from single cells were combined. To address this, we newly adopted a fresh paradigm in this test. We formed two groups for analysis: single cell‐derived PSC‐MPCs (sPSC‐MPC‐SCDs) including SCNT‐ or hES cell‐derived MPCs (sSCNT‐hPSC‐MPC‐SCDs or sES‐MPC‐SCDs) and combination cell‐derived PSC‐MPC‐SCDs (cPSC‐MPC‐SCDs) containing SCNT‐ or hES‐MPC‐SCDs (cSCNT‐hPSC‐MPC‐SCDs or cES‐MPC‐SCDs). First, all hPSC‐MSCs had also an exponential proliferation index and underwent more PDs, regardless of the single‐cell group or combination group. All hPSC‐MPCs were markedly increased in their cell numbers by greater than 1 × 10^17^‐fold within 75 days compared with those of hBM‐MPCs. (in CHA‐hES15‐MPCs, 10^11^‐fold; in CHA‐hNT5‐MPCs, 10^14^‐fold). In the present study, we have found that hPSC‐MPC‐SCD did not show more proliferative capacity than hPSC‐MPC. However, although most hPSC‐MPC‐SCDs can undergo limited passaging up to approximately 14 passages, their proliferating capacity is unarguably outstanding, suggesting the great value in the concept of therapeutic cell numbers.

Next, to examine whether these proliferative hPSC‐MPCs were safe from teratomas, we carried out the teratoma formation test. hPSC‐MPCs were injected into the testis of immunocompromised mice and were observed for 3 months. As shown in Figure 3I, all hPSC‐MPC‐SCDs did not produce teratomas (Figure 3I). However, teratomas or teratomas with cysts were clearly formed in parent CHA‐hES15 and CHA‐hNT5 cells. Based on the present data, we found that our safe and productive protocol for MPC differentiation from SCNT‐hPSCs can generate clinically applicable MPCs.

### 3.3 Establishment of a murine model of AS and inhibition of pro‐inflammatory factors in mice treated with SCNT‐MPC‐SCD

**Figure 4:**
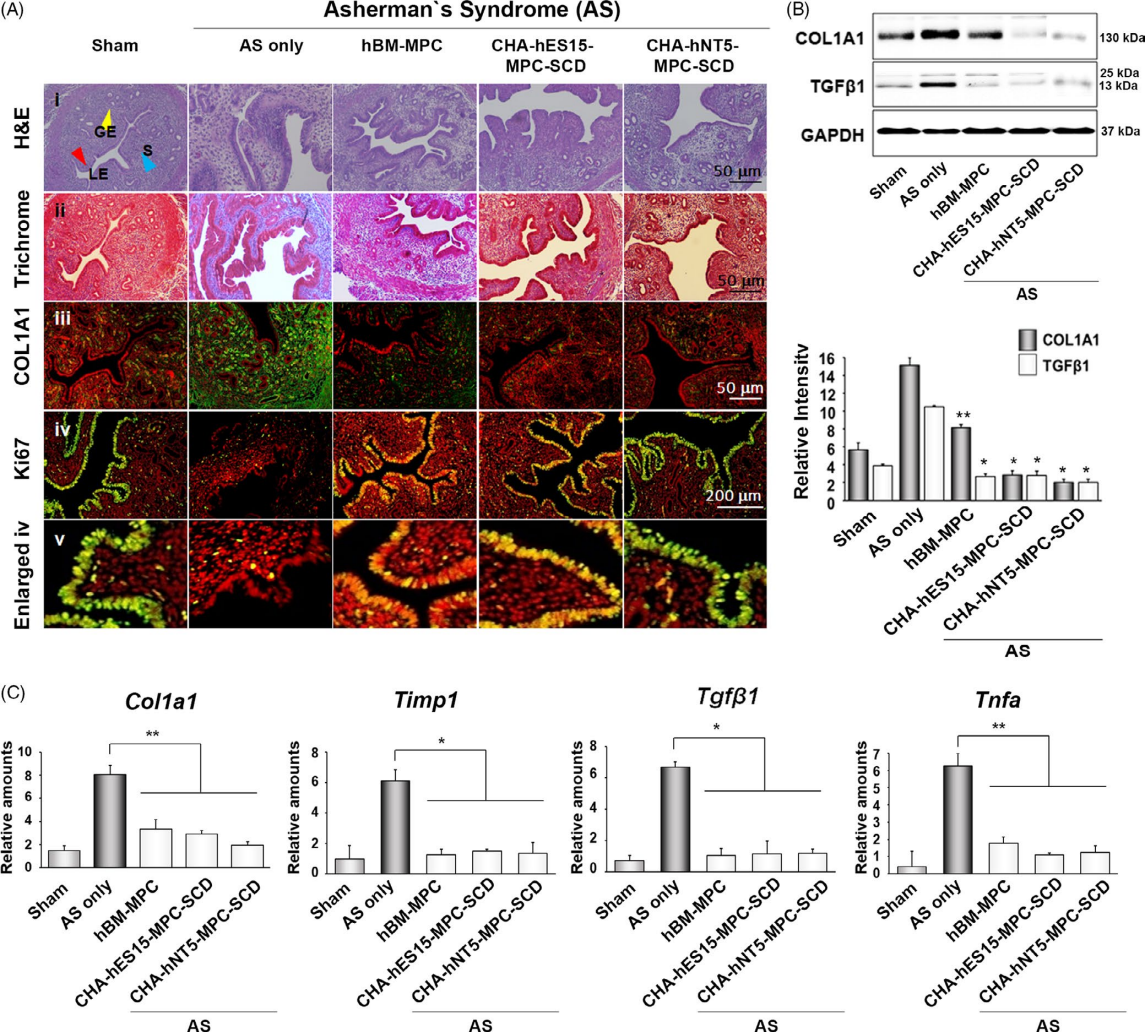
Induction of Asherman's syndrome (AS) in a murine model and inhibition of pro‐inflammatory factors in the hPSC‐MPC‐SCD‐treated group. (A) Immunohistochemistry showing the uterus in the AS‐disrupted stroma and luminal and glandular epithelia. Trichrome staining and COL1A1‐positive staining were very strong in the AS model, depicting fibrosis with abundant collagen. The enlarged panel shows the luminal epithelium with arrested proliferation, as confirmed by Ki‐67‐negative cells in the uterus. However, luminal and glandular cells were reinstated in the uterus, as similar to wild‐type, by MPC therapy. The red, yellow and blue arrow heads indicate the luminal epithelium (LE), glandular epithelium (GE) and stroma (S), respectively. Scale bar, 50 μm. (B) The expression levels of the COL1A1 and TGFß1 proteins were increased in the AS model and decreased by hPSC‐MPC‐SCDs, implying effective suppression of pro‐inflammatory factors by functional MPCs. The data are presented as the means ± SE from at least three experiments. Asterisks depict statistically significant differences compared with the AS model (**P < 0.05, *P < 0.01). (C) Consistent with the levels of proteins, the PCR data showed that pro‐inflammatory factors were highly increased in the AS model, but were significantly decreased by MSC therapy. The data are presented as the means ± SE from at least three experiments. Asterisks depict statistically significant differences compared with the AS model. Two independent experiments were performed (**P < 0.05, *P < 0.01).

We next sought to elucidate whether transplanted SCNT‐hPSC‐MPC‐SCDs can function as highly efficient therapeutic sources under pathologic condition. Similar with protocol by Alawadhi et al,^19^ we established the AS mouse model and prepared stem cells were directly injected into uterus after incision in anaesthetized mice. No remarkable difference in H&E staining was detected among the wild‐type, hBM‐MPC‐treated, both hPSC‐MPC‐treated groups, suggesting morphologic recovery into normal endometrium. However, the uteri of AS displayed a low frequency of small‐sized glandular epithelium in the stroma and a low density of cells in the stroma, implying fibrosis (Figure 4Ai). In trichrome staining, a blue‐coloured region presenting fibrosis was also displayed in the AS uterus. However, the MPC‐treated groups seemed to be recovered, shown as a reddish colour in the stroma, implying cellular fidelity (Figure 4Aii). Additionally, aberrant expression of COL1A1 was detected in the AS uterus (Figure 4Aiii) likely due to abundant collagen fibres and cell proliferative arrest, similar no previous pathogenic events.^27^ Additionally, single‐layered luminal epithelial cells, which are arrested by AS induction and are located on surface of endometrium, were highly increased by stem cell therapy (Figure 4Aiv). The proliferation of luminal epithelial cells was shown by an enlarged panel with nuclei (Figure 4Av). Fibrosis is closely involved with the failure of embryo implantation due to a defective endometrium.^28^ To investigate whether transplanted SCNT‐hPSC‐MPC‐SCDs contributed to fibrosis by the suppression of pro‐inflammatory factors in the uterus, Western blotting was carried out using pro‐inflammatory‐related factors TGFß1 and COL1A1. As shown in Figure 4B, proteins were dramatically decreased in hPSC‐MPC‐SCD‐treated groups. Consistent with the protein levels, the transcript levels in the wild‐type uterus were rarely expressed compared with those in the AS uterus, showing attenuation of fibrosis by reduced expression of pro‐inflammatory‐related genes. The expression levels of *Tgfß1, Tnfα*, *Timp1* and *Col1a1* were highly increased in the AS uterus by at least 3.7‐fold in CHA‐hES15‐MPC‐SCDs and CHA‐hNT5‐MPC‐SCDs (Figure 4C), showing congruous movement of RNA and proteins in cell therapy. However, signals for X‐ and Y‐chromosome probes in the AS‐induced mouse uterus were not detected at day 7 after transplantation of MPCs, and it may suggest that transplanted human cells could be disappeared (Figure S4).

### 3.4 Regeneration of damaged uteri in AS by SCNT‐MPC‐SCD transplantation via enhanced angiogenesis from host cells

**Figure 5:**
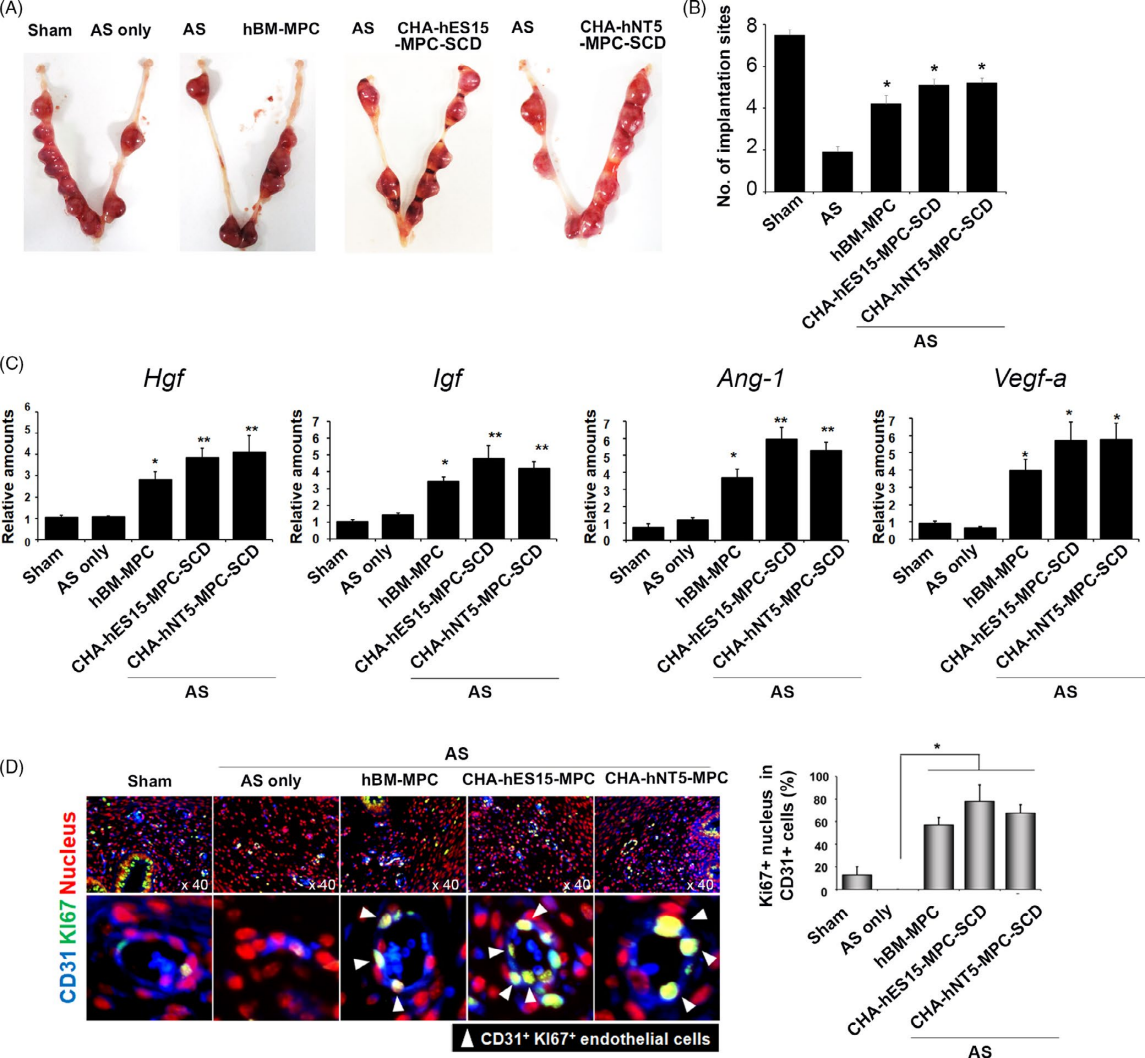
Impaired implantation in the AS uterus can be ameliorated in the hPSC‐MPC‐SCD‐treated group by angiogenesis of host cells. (A) Assessment of implantation failure using the AS model. (B) The average number of pups is shown. Statistical analysis of (A), presented as the means ± SE from at least three experiments. Asterisks depict statistically significant differences compared with implantation from the AS model. Graphs from 2 independent experiments are shown. N = 6 per group. (all group except AS = 6 horns, AS = 24 horns). (**P < 0.05, *P < 0.01). (C) Host murine cells upregulate the angiogenic factors, including *Hgf, Igf, Ang‐1* and *Vegf‐a. *To determine whether the high cytokine levels were derived from human or murine cells, real‐time PCR was performed using human‐ and mouse‐specific primers. Most cytokines were murine, not human, and were highly sustained at day 14. Individual values were normalized to *rPL7. *The data for B and C are presented as the means ± SE from at least three experiments. Asterisks depict statistically significant differences compared with implantation from the AS model (**P < 0.05, *P < 0.01). (D) The capillary density was measured by counting Ki‐67^+^ (green) and CD31^+^ cells (blue). DAPI was used to detect nuclei (red colours). Statistical analyses of the panels and values are presented as the mean ± SE from at least randomly selected 15 fields from four mouse heads per group. Two independent experiments were performed. N = 6 per group. (all group except AS = 6 horns, AS = 24 horns). (**P < 0.05, *P < 0.01) Magnification, ×40.

Following this evidence, we examined the functional improvement of the injured uterus with AS for pregnancy by SCNT‐hPSC‐MPC‐SCD treatment. The numbers of implanted embryos were counted in the uterus on day 12 of pregnancy. As expected, all Sham uterine horns with no injury had ~7 implantation sites (IS)/uterine horn. In AS uterine horns, ~2 IS were observed, while ~5 IS were observed in the hPSC‐MPCs‐treated horns (Figure 5A). However, the number of IS in all hPSC‐MPC did not reach that in the Sham control (Figure 5B); the numbers were significantly higher than those in the AS group, suggesting the therapeutic effects of hPSC‐MPCs in AS.

The suboptimal number of IS in the uterus with AS could be caused by insufficient angiogenesis because blood vessels play a role to deliver nutrients and growth litters. Moreover, it is well known that MPCs function to increase angiogenesis through paracrine‐angiogenic factors.^29^, ^30^ To prove this, we determined the capillary density in both hPSC‐MPC‐SCD‐treated groups in the AS condition where the formation of new blood vessels is needed to prevent fibrosis.^31^ Intriguingly, we found that angiogenic factors were highly increased in the uterus receiving MPCs, even 7 days after therapy. Compared with genes of the AS uterus, mouse angiogenic factors, including *Hgf, Igf, Ang‐1 *and *Vegf‐a*, in the MSC‐treated group were significantly elevated in recipient tissues, while human genes show no expression, implying the activation of recipient cells by transplanted SCNT‐hPSC‐MPC‐SCDs (Figure 5C). Previously, Cervello et al^28^ addressed the importance of blood vessels in regeneration of the uterus by showing the engraftment of stem cells near blood vessels. To further determine the existence of angiogenesis in the AS uterus at 7 days after MPC transplantation, we subsequently quantified the number of proliferating endothelial cells in recipient uteri by co‐immunofluorescence staining for CD31 (a representative marker for endothelium) and Ki67 (a cell proliferation marker). As shown in Figure 5D, proliferation of endothelial cells was similar between CHA‐hES15‐MPCs and CHA‐hNT5‐MPCs but was not observed in the AS group (in sham: 12.9 ± 7.1%; in AS: 0 ± 0%; in hBM‐MPCs: 57.0 ± 6.5%; in CHA‐hES15‐MPC‐SCDs: 77.7 ± 14.9%; in CHA‐hNT5‐MPC‐SCDs: 67.5 ± 7.5%) (Figure 5D). These data directly indicate that SCNT‐hPSC‐MPCs dramatically increased the angiogenic effects in the AS uterus, resulting in improved uterine function for pregnancy.

## 4 DISCUSSION

In the present study, we first established single cell‐derived clonally expanded MPCs from human pluripotent stem cells using somatic cell nuclear transfer technology. The salient findings for these MPCs are as follows: First, to produce MPCs, SCNT‐hPSC‐derived MPCs comprised two phases: the initial differentiation phase of MPCs and single cell‐derived homogeneous clonal expansion of MPCs with safety from teratomas. Second, we showed that transplanted SCNT‐hPSC‐MPCs into the uterus with AS induced a significant increase in angiogenic factors during repair and remodelling, resulting in better pregnancy outcomes and suggesting the functionality of SCNT‐hPSC‐MPCs. Third, we uncovered that the therapeutic benefits are attributed primarily to a host‐dependent mechanism by the injected functional SCNT‐hPSC‐derived MPCs, which serve as a favourable milieu for angiogenesis and regeneration of the AS uterus. Finally, we showed cumulative cell numbers, high population doublings, and no teratomas from the generated MPCs, which resemble hESC‐MPCs. Therefore, our protocol to guarantee the generation of functional SCNT‐hPSC‐MPCs from single MPCs following initial differentiation makes it possible to have an overwhelming number of the cells in a short period of time. Although the generation of hPSCs using SCNT has technical difficulties, somatic cell reprogramming technology is emerging as a promising tool due to a low risk of immune rejection^32^ as well as the presentation of the full genome from a patient, who displays genomic aberrant symptom. Additionally, several reports continuously showed that iPSCs show detailed differences at the molecular levels; gene expression, genomic integrity and DNA methylation have been continuously reported for PSCs compared with those of ESCs.^33^, ^34^, ^35^ Because SCNT technology can bring adult cells back to the embryonic stage. It is similar to the classical ESC. This rejuvenate advantage is expected to emphasize that SCNT‐hPSC will play an important role in degenerative medicine as well as regenerative medicine. Also, PSC line cells derived from SCNT could be used for the study of their pathologic mechanisms and applied clinically. Thus, we generated PSCs using SCNT that differentiated into MPCs and investigated the function of SCNT‐hPSC‐MPCs in vivo and in vitro. The proliferative rate and PDs of the SCNT‐hPSC‐MPC‐SCDs were significantly higher and longer than those of BM‐MPCs, with a doubling occurrence of approximately 35.6 hour. These cells proliferated consistently, reached a peak at P30 (data not shown) and then gradually decreased, sustaining a high proliferative activity. Additionally, all homogeneous clonally expanded MSCs showed no teratoma formation. To attain the therapeutic dose, optimized MPCs in early passages without the senescent phase should be prepared in large numbers depending on the disease. SCNT‐hPSC‐MPC‐SCDs have solved these problems by demonstrating safe cell proliferation and no teratoma formation.

AS is relevant to the damage of endometrial cells of the uterus where angiogenesis regularly occurs as part of the menstrual cycle. VEGFR‐2 is main protein in blood endothelium, and its inhibition effectively suppresses non‐alcoholic steatohepatitis accompanied by fibrosis.^36^ Fibrosis in AS was successfully recovered by both SCNT‐hPSC‐MPC‐SCDs and hESC‐MPC‐SCDs, evoking angiogenesis. Recently, Santamaria et al^37^ performed a pilot study that human autologous CD133^+^ stem cells can function as a promising therapeutic option in AS patients and completed clinical trials with phase 4. (ClinicalTrials.gov. NCT02144987). Also, mesenchymoangioblast‐derived mesenchymal stem cells were used in clinical trials with phase 1 (ClinicalTrials.gov.NCT02923375). Human stem cells are regarded as a therapeutic cell sources in treating human disease. Consistent with this, we found de novo cell sources from human PSCs and addressed the feasibility of MPCs for cell therapy in AS.

In conclusion, SCNT‐hPSC‐MPC‐SCDs can contribute to rescue fibrosis in the uterus of AS mice, leading to successful implantation by encouraging the inhibition of pro‐inflammatory factors and promoting angiogenic factors from host cells. MPCs generated from PSCs using SCNT show high cellular proliferation and differentiation activities, without teratomas. These properties of SCNT‐hPSC‐MPC‐SCDs are similar to those of ESCs and overcome the limitation of adult BM‐MPCs. In addition, production of these novel cells suggests that therapy using SCNT‐hPSC‐MPCs can be an innovative modality for specific patients with an incurable disease in regenerative medicine.

## CONFLICTS OF INTEREST

The authors indicate no potential conflicts of interest.
