# Supplementary material for: Single cell‐derived clonally expanded mesenchymal progenitor cells from somatic cell nuclear transfer‐derived pluripotent stem cells ameliorate the endometrial function in the uterus of a murine model with Asherman’s syndrome
Source: Cell Prolif. 2019 Mar 21;52(3):e12597. doi: 10.1111/cpr.12597 (PMC6536448; doi:10.1111/cpr.12597)
Supplement: Supplementary file 1 [file CPR-52-e12597-s001.docx]

Single cell-derived clonally expanded mesenchymal progenitor cells from somatic cell nuclear transfer-derived pluripotent stem cells ameliorate the endometrial function in the uterus of a murine model with Asherman’s syndrome

Sung-Min Jun^1,*^, Mira Park^2,*^Ji Yoon Lee^2,*^, Sookyung Jung^1^, JeoungEun Lee^1^, **Sung** Han Shim^2^,Haengseok Song^2,#^, and Dong Ryul Lee^1,2, #^

^1^ CHA Stem Cell Institute, CHA University, Seongnam, Gyounggi-do 13488, Korea

^2^ Department of Biomedical Science, CHA University, Seongnam, Gyounggi-do 13488, Korea

^*^ These authors contributed to this work equally

^#^To whom co-correspondence should be addressed: e-mail: [drleedr@cha.ac.kr](mailto:drleedr@cha.ac.kr) and [hssong@cha.ac.kr](mailto:hssong@cha.ac.kr)

**Supplemental information**

Supplemental MATERIALS AND METHODS

**Quantitative RT-PCR (qRT-PCR)**

Isolation of total RNA and cDNA synthesis were performed as previously described. For conventional PCR, Reverse transcription (RT)-PCR was then performed using 1μl cDNA which was amplified for 30 cycles at 57-61°C using the proper primers. Samples were separated using a 1.5% agarose gel and visualized using a UV transilluminator. For real-time PCR, SYBR green real-time PCR master mixes was used according to the manufacturer`s instructions. Relative mRNA expression of target genes was calculated using the comparative CT method. All target genes were normalized to *GAPDH* in multiplexed reactions performed in triplicate. Differences in CT values were calculated for each target mRNA by subtracting the mean value of *GAPDH* (relative expression=2^‐ΔCT^). Information of primer sets used in this study is listed in supplementary table 1.

**Immunocytochemistry and measurement of blood vessel capillary density**

Differentiated cells were initially fixed with 2% PFA for 5 min at room temperature. After washing with PBS, the cells were blocked with 5% serum and were subjected to staining with primary antibodies, followed by incubation with secondary antibodies. To examine the differentiation capacity of MPCs, Oil red O, Alizarin Red S, and Alcian Blue staining assays were performed. Angiogenic events were assessed using the Ki67 antibody (Abcam; ab16667; 1:200), CD31 (BD Biosciences; 553370, 1:200), followed by counterstaining with DAPI. Cells positive for FITC-CD31, Ki67 and DAPI were counted in uterus. For statistical analysis, the capillary density (number of CD31^+^ blood vessels and Ki67^+^ nuclei) was calculated from at least 10 randomly selected fields using 8 uteri. All the samples were visualized under an inverted or fluorescence microscope counterstained with Meyer’s hematoxylin or DAPI, respectively. To confirm the fibrosis of AS, hematoxylin and eosin (H&E), trichrome and COL1A1 (Novus Biologicals, NB600-408, 1:200) staining was performed after fixation.

**Western blotting**

Uterine samples were homogenized with a Polytron homogenizer and protein extracts were prepared by lysing cells in PRO-PREP Protein Extraction Solution (iNtRON Biotechnology) and 1 × [phosphatase](https://www.sciencedirect.com/topics/biochemistry-genetics-and-molecular-biology/phosphatase) Inhibitor (Roche Applied Sciences). Samples (20 μg) were separated by [SDS-PAGE](https://www.sciencedirect.com/topics/biochemistry-genetics-and-molecular-biology/polyacrylamide-gel-electrophoresis) (8–10%) and transferred to nitrocellulose-membrane (Bio-Rad). After transfer, the membranes were subjected to [Western blotting](https://www.sciencedirect.com/topics/biochemistry-genetics-and-molecular-biology/western-blot) with anti-COL1A1 (Novus Biologicals, NB600-408,1:1000), anti-TGF-beta1 (Santacruz, sc-130348, 1:1000), and anti-GAPDH (Cell signaling, #2118,1:2000) antibodies. [Immunoreactive](https://www.sciencedirect.com/topics/biochemistry-genetics-and-molecular-biology/immunoassay) bands were detected using the Immune-Star Western™ [Chemiluminescence](https://www.sciencedirect.com/topics/biochemistry-genetics-and-molecular-biology/chemiluminescence) Kit (Bio-Rad). The chemiluminescence signal was detected using the ChemiDOC™ XRS + system (Bio-Rad).

**Flow cytometry**

Cells were briefly resuspended in 100 μl of rinsing buffer and were incubated with antibodies. We used allophycocyanine (APC)-conjugated mouse anti-human CD29 (559883; BD Pharmingen), APC-conjugated mouse anti-human CD44 (559942; BD Pharmingen), APC-conjugated mouse anti-human CD90 (561971; BD Pharmingen), and APC-conjugated mouse anti-human CD105 (562408; BD Pharmingen) for MPC markers. APC-conjugated mouse anti-human CD34 (555824; BD Pharmingen), APC-conjugated mouse anti-human CD45 (555485; BD Pharmingen), phycoerythrin (PE)-conjugated mouse anti-human Tra-1-60 (560193; BD Pharmingen), and APC-conjugated mouse anti-human SSEA4 (FAB/435A; R&D systems) as stem and hematopoietic cell markers were used to examine differentiated cells. Regarding secondary antibodies, proper isotype-matched IgG and unstained controls were used. After washing, the cells were analyzed using a FACSCalibur flow cytometer equipped with Cell Quest software (BD Biosciences, San Diego, CA, USA).

|  |  |
| --- | --- |

**Karyotype**

Karyotyping Cells were received at approximately 80% confluence in culture dish. Colcemid was applied to the cells to arrest mitotic division and cells were harvested by standard hypotonic treatment after fixation. Poly-L lysine coated slides were prepared and hybridized to observe G-banding in chromosome. Post hybridization washes were performed according to the protocols provided by the manufacturer and established in CHA laboratory. Metaphase cells were analyzed to confirm normal karyotype in differentiated cells.

**Teratomas formation assay**

Protocols for the use of animals in present all experiments were approved by the Institutional Animal Care and Use Committee of CHA University (IACUC) and animal procedures were performed in accordance with approved guidelines and regulation. Fully differentiated single cell derived-clonal expanded MPCs from hPSCs, and the parent hPSCs line cells were adjusted at 1x 10^6^ cells and injected intratesticularly into male immunocompromised mice. Mice were sacrificed at 3 months after initial injection. Teratomas were extracted, and assessed existence of teratomas in gross examination.

**Colony-Forming Assay**

Colony formation assay was performed to evaluate the ability of differentiated MPCs in terms of early-passage and late-passage. Brieﬂy, aliquots of 500 cells/78.5 cm^2^ were cultured at 37°C and 5% CO^2^ for 14 days with medium change twice a week. Cells were then ﬁxed with 4% paraformaldehyde for 15 minutes, followed by washing using ddH2O for twice and stained with 0.1% crystal violet for 30 minutes at room temperature. Cell colony counting was performed on a microscope.

**FISH and Immunohistochemical Analysis**

To investigate the existence of injected Y chromosome-positive MPCs in uterus tissues, FISH staining was performed with the chromosome enumeration probes (CEP) Y- and X-DNA probes kit (Abbott Molecular, Des Plaines, IL). Tissue was ﬁxed in 4% paraformaldehyde and dehydrated in an ethanol gradient. The sample was embedded in parafﬁn and sectioned into 4-μm-thick sections. Tissue sections were denatured in 70% (vol/vol) formamide/2x standard sodium citrate (SSC) at 72°C for 2.5 minutes, quenched in ice-cold 70% (vol/vol) ethanol, and dehydrated in a cold ethanol series for 2 minutes per solution. The probe was denatured at 75°C for 10 minutes and then sealed under cover slips with rubber cement. Hybridization was performed overnight at 37°C in a humidiﬁed chamber. After hybridization, the sections were washed for 10 minutes with 50% (vol/vol) formamide/ 2x SSC at 42°C and placed in 4x SSC with 0.1% Nonidet P40 for 5 minutes; this was followed by air drying in the dark and counterstaining with 4´,6-diamidino-2-phenylindole (DAPI; Sigma, St. Louis, MO). To detect cells expressing X,Y-specific DNA sequence, whole tissue sections were examined under confocal microscope (Zeiss LSM880).

**
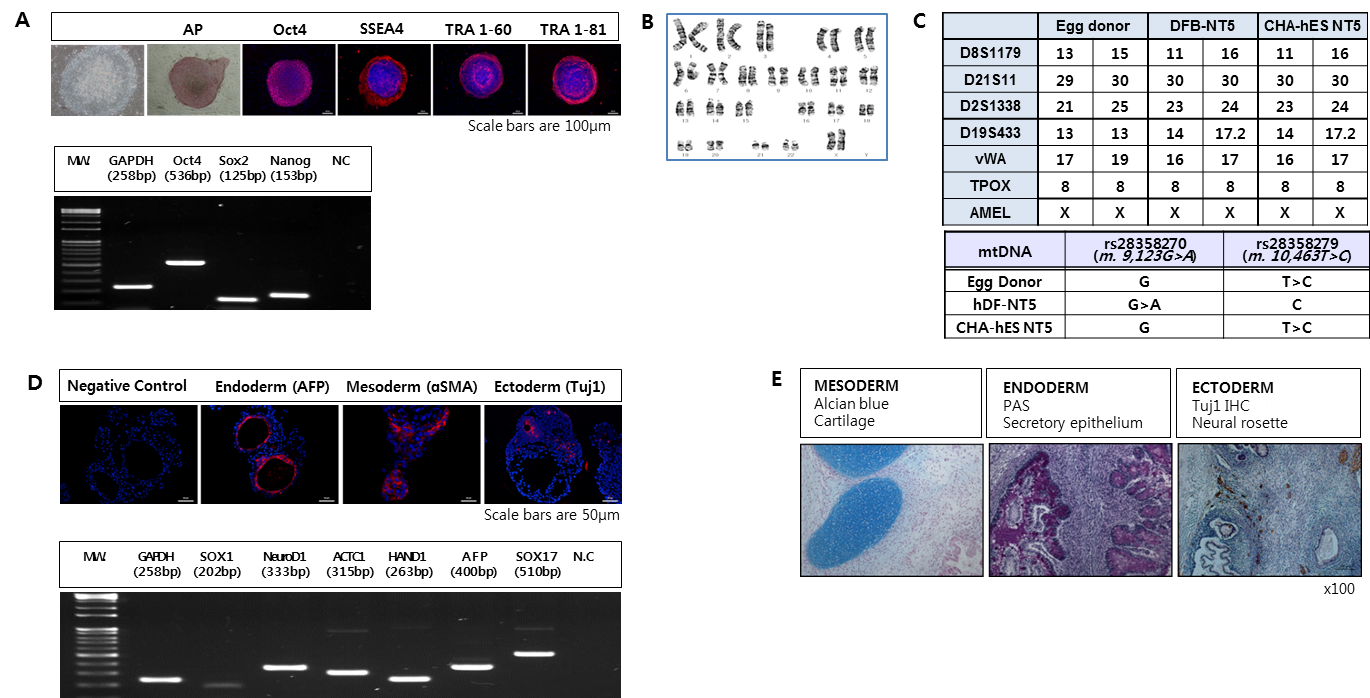
**

Supplementary Figure 1. Characteristic of CHA-hESNT5 pluripotent stem cell line using somaticcell nuclear transfer.(A) Immunocytochemistry and RT-PCR showed stableES colony morphology and expression of marker for pluripotency. CHA-hES NT5 expressed phosphatase activity and ES cell markers including OCT-4, SSEA-4, TRA 1-60, TRA-1-81 in protein levelsas well as Oct-4, Nanog, and Sox-2 in mRNA levels. (B) Normal karyotyping results was shown by G-banding (46,XX). (C) Tables showed the information for nuclear DNA and mitochondrial DNA genotyping. It clearly showed that there was no contribution of oocyte nuclear DNA to SCNT-ESC, and mt-DNA were derived from the oocyte. (D) The expressions of markersfor differentiation in embryiod bodies derived from CHA-hES NT5 were addressed by RT-PCR and immunohistochemistry. (E) The immunohistochemistry provided the capacity for teratomas formation from CHA-hES NT5 by lineage marker expressions.


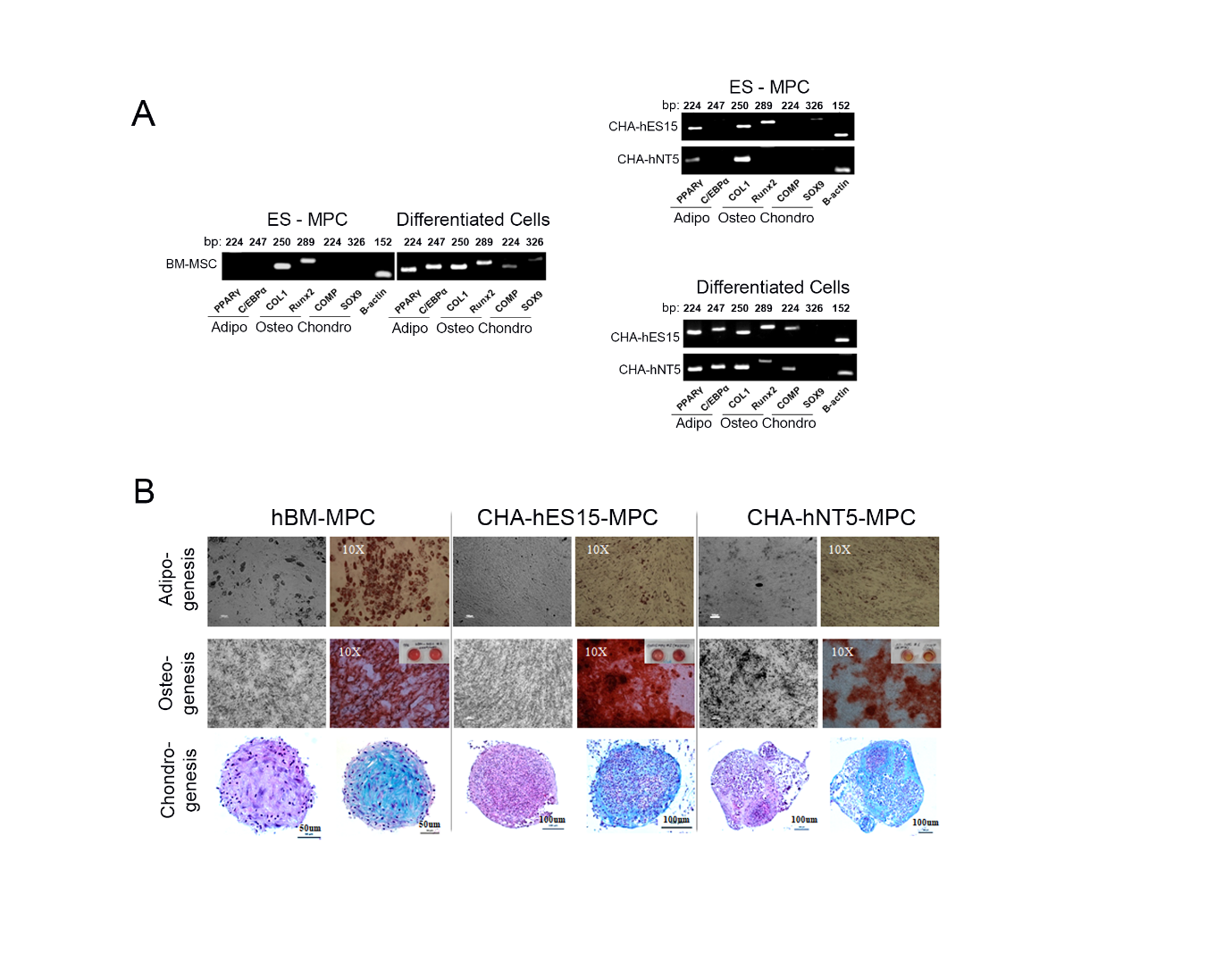


Supplementary Figure 2. Potential of differentiation for multilineage cells from SCNT-hPSC-MPCs. (A) In multiple lineage differentiation,The *PPARγ,C/EBPα* for adipocytes*, COL1, RUNX2* for osteocytes*,* and *COMP*, *SOX9* for chondrocytes in hPSC-MPCs was detected by differentiation. All transcripts were expressed in multilineage cell differentiation. (B) To confirm their maturation, hPSC-MPCs using standard differentiation conditions for adipogenesis, osteogenesis, and chondrogenesis were differentiated, however, adipogenesis was not fully differentiated in MPC status, suggesting the not full maturation of hPSC-MPCs into adipocyte, osteocyte and chondrocyte.


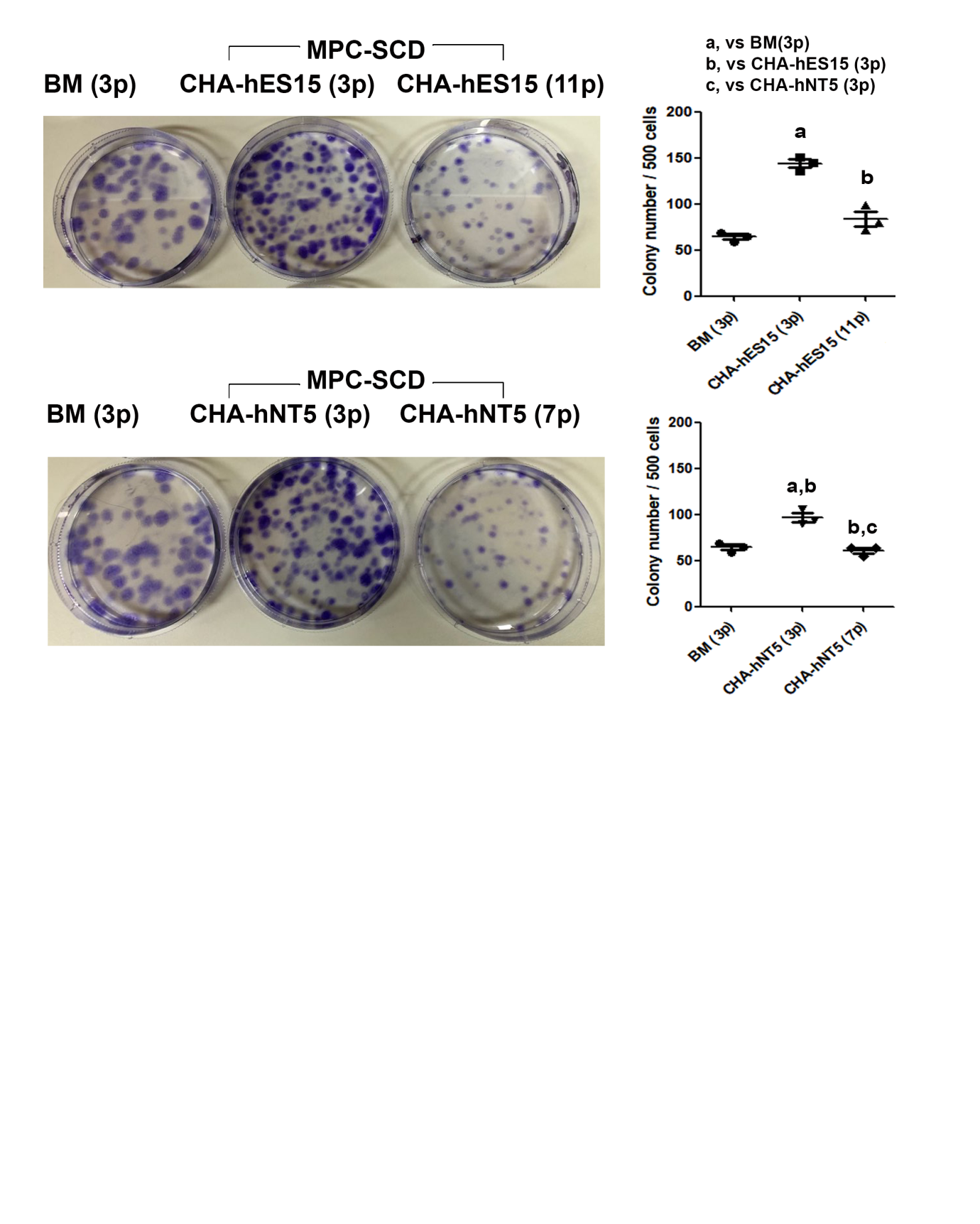
Supplementary Figure 3. Colony forming assay was performed using various stages of MPC-SCD (CHA-hES15 and CHA-hNT5 vs. hBM-MPCs). Quantiﬁcation of the colony numbers for early- and late-passage differentiated MPCs was performed with early passaged human BM-MPCs (control). Cells were stained with crystal violet (left panel) and colony numbers were counted (right panel). Both colony from early passaged CHA-hES15 MPCs and CHA-hNT5 MPCs was significantly increased compared to that of human BM-MPCs. A number of colonies from late passaged CHA-hES15 MPCs and CHA-hNT5 MPCs were remarkably lower than in early passaged CHA-hES15 MPCs and CHA-hNT5 MPCs. Data is presented as the mean±SEM.


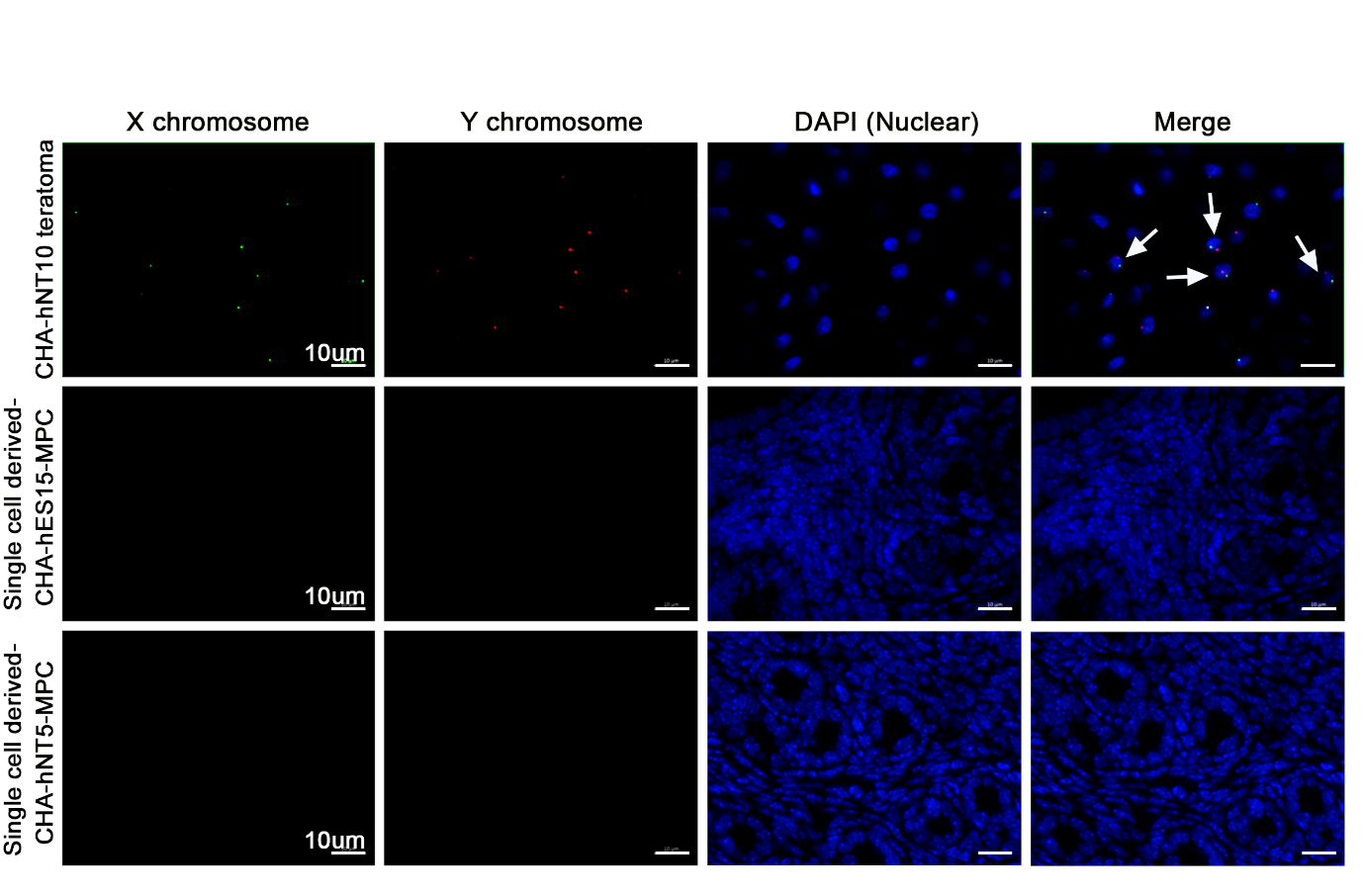


Supplementary Figure 4. Signals for X- and Y-chromosome probes in the AS-induced mouse uterus were not detected at day 7 after transplantation of MPCs, and it may suggest that transplanted human cells could be disappeared. Meanwhile, X-,Y-probe in teratoma tissue as a positive control was clearly detected (white arrows). Scale bars represent 10 μm.

**Supplementary Table 1. Primers and probes for quantitative RT-PCR**

|  | | | | |  | | | | |  |
| --- | --- | --- | --- | --- | --- | --- | --- | --- | --- | --- |
|  | |  |  | |  |  | |  | | |
| Genes |  | | Primers and probes (5'-3') | Tm | | | Cycle | | Size (b.p.) |  |
| *human β-actin* | | Forward | TGAAGTGTGACGTGGACATC | 58 | | | 30 | | 152 |  |
|  | | Reverse | GGAGGAGCAATGATCTTGAT |  |  |  |  |  |  |  |
| *human PPARγ* | | Forward | TGTCTCATAATGCCATCAGGTTTG | 57 | | | 30 | | 224 |  |
|  | | Reverse | GATAACGATGGTGATTTGTCTGTT |  |  |  |  |  |  |  |
| *human C/EBPα* | | Forward | GCAAACTCACCGCTCCAATG | 57 | | | 30 | | 247 |  |
|  | | Reverse | TTAGGTTCCAAGCCCCAAGTC |  |  |  |  |  |  |  |
| *human COL-1* | | Forward | AGAACATCACCTACCACTGC | 57 | | | 30 | | 250 |  |
|  | | Reverse | ATGTCCAAAGGTGCAATATC |  |  |  |  |  |  |  |
| *human RUNX2* | | Forward | CCGCACGACAACCGCACCAT | 61 | | | 30 | | 289 |  |
|  | | Reverse | CGCTCCGGCCCACAAATCTC |  |  |  |  |  |  |  |
| *human COMP* | | Forward | AACGCTGAAGTCACGCTCAC | 60 | | | 30 | | 224 |  |
|  | | Reverse | GGTAGCCAAAGATGAAGCCC |  |  |  |  |  |  |  |
| *human SOX9* | | Forward | TTCATGAAGATGACCGACGA | 60 | | | 30 | | 326 |  |
|  | | Reverse | CACACCATGAAGGCGTTCAT |  |  |  |  |  |  |  |
| *human OCT4* | | Forward | AGT GAG AGG CAA CCT GGA GA | 60 | | | 30 | | 110 |  |
|  | | Reverse | ACA CTC GGA CCA CAT CCT TC |  |  |  |  |  |  |  |
| *human SOX2* | | Forward | AGC TAC AGC ATG ATG CAG GA | 60 | | | 30 | | 125 |  |
|  | | Reverse | GGT CAT GGA GTT GTA CTG CA |  |  |  |  |  |  |  |
| *human NANOG* | | Forward | TGA ACC TCA GCT ACA AAC AG | 60 | | | 30 | | 153 |  |
|  | | Reverse | TGG TGG TAG GAA GAG TAA AG |  |  |  |  |  |  |  |
| *human GAPDH* | | Forward | AGAAGGCTGGGGCTCATTTG | 60 | | | 35 | | 258 |  |
|  | | Reverse | AGGGGCCATCCACAGTCTTC |  |  |  |  |  |  |  |
| *human HGF* | | Forward | CAATAGTCAATTTAGACCATCCCGTAAT | 60 | | | 35 | | 129 |  |
|  | | Reverse | CGTGTTGGAATCCCATTTACAA |  |  |  |  |  |  |  |
| *human IGF* | | Forward | CCATGTCCTCCTCGCATCTC | 60 | | | 35 | | 128 |  |
|  | | Reverse | CGTGGCAGAGCTGGTGAAG |  |  |  |  |  |  |  |
| *human ANG-1* | | Forward | CAGAAAACAGTGGGAGAAGATATAACC | 60 | | | 35 | | 105 |  |
|  | | Reverse | TGCCATCGTGTTCTGGAAGA |  |  |  |  |  |  |  |
| *human VEGF-A* | | Forward | GTGCCCACTGAGGAGTCCA | 60 | | | 35 | | 106 |  |
|  | | Reverse | TCCTATGTGCTGGCCTTGGT |  |  |  |  |  |  |  |
| *mouse Col1a1* | | Forward | CTG GCG GTT CAG GTC CAA T | 60 | | | 26 | | 141 |  |
|  | | Reverse | TTC CAG GCA ATC CAC GAG C |  |  |  |  |  |  |  |
| *mouse Mmp2* | | Forward | GCG ATG TCG CCC CTA AAA CAG | 60 | | | 28 | | 265 |  |
|  | | Reverse | CTG TAT GTG ATC TGG TTC TTG TCC |  |  |  |  |  |  |  |
| *mouse Timp1* | | Forward | GGG TTC CCC AGA AAT CAA CGA G | 60 | | | 28 | | 139 |  |
|  | | Reverse | ACA GAG GCT TTC CAT GAC TGG GGT G |  |  |  |  |  |  |  |
| *mouse Tgf-beta1* | | Forward | GTG AAA CGG AAG CGC ATC GAA G | 60 | | | 30 | | 193 |  |
|  | | Reverse | CAT AGT AGT CCG CTT CGG GCT CC |  |  |  |  |  |  |  |
| *mouse Tnfaa* | | Forward | CTGAACTTCGGGGTGATCGG | 60 | | | 28 | | 122 |  |
|  | | Reverse | GGCTTGTCACTCGAATTTTGAGA |  |  |  |  |  |  |  |
| *mouse Rpl7* | | Forward | TCA ATG GAG TAA GCC CAA AG | 60 | | | 22 | | 246 |  |
|  | | Reverse | CAA GAG ACC GAG CAA TCA AG |  |  |  |  |  |  |  |
| *mouse Hgf* | | Forward | CTGACCCAAACATCCGAGTTG | 60 | | | 35 | | 125 |  |
|  | | Reverse | TTCCCATTGCCACGATAACAA |  |  |  |  |  |  |  |
| *mouse Igf* | | Forward | TGCTTCCGGAGCTGTGATCT | 60 | | | 35 | | 125 |  |
|  | | Reverse | CGGGCTGCTTTTGTAGGCT |  |  |  |  |  |  |  |
| *mouse Ang-1* | | Forward | GGGACAGCAGGCAAACAGA | 60 | | | 35 | | 110 |  |
|  | | Reverse | TGTCGTTATCAGCATCCTTCGT |  |  |  |  |  |  |  |
| *mouse Vegf-a* | | Forward | GCAGGCTGCTGTAACGATGA | 60 | | | 35 | | 105 |  |
|  | | Reverse | GCATGATCTGCATGGTGATGTT |  |  |  |  |  |  |  |
